# Extensively drug-resistant *Acinetobacter baumannii* complex (ABC) colonization and clinical outcomes in neonates in a tertiary hospital, South Africa: 2020–2022

**DOI:** 10.1017/ash.2026.10879

**Published:** 2026-10-10

**Authors:** Gugulethu Julia Phambane, Sinenhlanhla Ndzabandzaba, Joy Fredericks, Rispah Chomba

**Affiliations:** 1 Department of Clinical Microbiology and Infectious Diseases, School of Pathology, Faculty of Health Sciences, University of the Witwatersrand, Johannesburg, South Africa; 2 Department of Paediatrics, Faculty of Health Sciences, University of the Witwatersrand, Johannesburg, South Africa

## Abstract

**Background:**

Extensively drug-resistant *Acinetobacter baumannii* (XDR-AB) is a significant cause of healthcare-associated infections, particularly in neonatal intensive care units. This study aimed to determine the prevalence of XDR-AB colonization and the odds of subsequent invasive infection. The secondary objectives were to identify risk factors and in-hospital outcomes.

**Methods:**

A retrospective study was conducted among 485 neonates admitted to Rahima Moosa Mother and Child Hospital in Johannesburg, South Africa, from June 1, 2020 to December 31, 2022, using laboratory data and medical records.

**Results:**

Among 485 neonates, 116 babies (23.9%) were colonized with XDR-AB, with a median colonization age of 4 days. A total of 32 neonates developed invasive infections. Invasive XDR-AB disease was significantly more common among colonized neonates (29 neonates (25%)) compared to 3 (0.81%) non-colonized neonates. In-hospital mortality was significantly higher in colonized neonates (12 (10.34%)) compared to non-colonized neonates (5 cases (1.36%)). Other in-hospital outcomes were not significantly associated with colonization status. Independent colonization risk factors were all affected by confounding.

**Conclusions:**

This study highlights the high prevalence of early colonization and subsequent invasive disease with its associated mortality in neonates. Screening of neonates for early colonization helps in empiric antibiotic choices for those found to be colonized.

## Introduction

Healthcare-associated infections (HAIs) pose a substantial burden, leading to prolonged hospital stays, limited treatment options, increased health costs, morbidity, and mortality.^1,2^ Neonates and critically ill patients are vulnerable due to their immature immune system response.^3^ Neonatal sepsis ranks as the third most common cause of neonatal mortality worldwide, with antimicrobial resistance (AMR) accounting for 31% – 63% of these deaths.^1,4^ Developing countries have higher HAI rates, especially in African neonatal intensive care units (NICUs), where *Acinetobacter baumannii* accounts for a large proportion of infections.^2^ Rising AMR limits effective treatment options, particularly in low- and middle-income countries (LMICs), highlighting the need for better understanding of resistance mechanisms and pathogen behavior.^5^

The *A. baumannii* complex is a WHO-declared priority pathogen that frequently causes NICU outbreaks in individuals under one year of age.^1,5–7^ South African surveillance from 2017 to 2019 revealed that at least 75% of *A. baumannii* were extensively drug resistant (XDR), with the highest burden in Gauteng Province.^7^ This Gram-negative coccobacilli microorganism consists of several species, which are commonly found in the water and soil, hospital environment, on the skin of healthy individuals and can colonize invasive devices without necessarily causing invasive disease.^5,7–9^ When infection occurs, ventilator-associated pneumonia and bloodstream infections are the most common invasive HAIs.^2,10^ Its ability to rapidly acquire AMR including high meropenem and colistin resistance rates of 88% and 4%, respectively, combined with environmental persistence complicates eradication, whereas novel agents (eg, ampicillin-sulbactam, sulbactam-durlobactam, and eravacycline) remain unaffordable in LMICs.^5,7,9,11,12^

The rate of extensively drug-resistant *A. baumannii* (XDR-AB) colonization among preterm neonates ranges from 12% to 21.4%, particularly in the first week of life.^3,13–15^ Key colonization risk factors include younger age, male sex, small gestational age, cesarean delivery, and respiratory distress requiring invasive ventilation.^10,11,13–15^

Notably, in a South African Pediatric Intensive Care Unit (PICU), approximately 57% of the neonates who were colonized remained so for the duration of their PICU stay, a finding associated with prolonged hospitalization.^10^ Colonization is postulated to precede infection and is a critical risk factor for subsequent invasive infection and poor prognosis.^11^ Gut colonization significantly increases the risk of bloodstream infections with XDR-AB (RR 3.6).^4^ Approximately 7.9% to 25% of colonized neonates in the NICU develop invasive disease, including bloodstream infections, compared to 5.8% in non-colonized neonates.^3,15,16^ A South African laboratory-based surveillance study reported XDR-AB-associated bloodstream infections in approximately 41% of infants, however, this study didn’t distinguish between colonized or non-colonized infants.^7^

Patients colonized with XDR-AB are more likely to develop invasive disease when they have factors such as invasive ventilation, previous antibiotics, renal replacement therapy, cardiovascular disease, and extended ICU stays.^3,10,11,13–15^

Khurana *et al.* found a postcolonization mortality risk of 26%, with cases of XDR-AB bloodstream infections preceded by colonization showing a high mortality rate of 75.^3^ However, the overall risk of mortality was higher in the colonized group at 6 months. Additionally, Perovic *et al.* reported that 36% of neonates infected with XDR-AB died during hospitalization.^7^ Mortality was significantly higher in infected neonates compared with those merely colonized (39% vs 9.5%, *P* = .01).^15^ Mortality risk was particularly high in the infected neonates with lower birth weight (mean range of 960g to 1685g) and younger age (mean 6 d).^3,7,14^

Preterm neonates are quickly colonized through maternal and environmental sources, and early detection of XDR-AB carriage is essential to implement infection-control measures, limit transmission, and identify neonates at risk of developing invasive infection.^3,6^ Studies exploring the relationship between HIV infection and *A. baumannii* acquisition or related mortality are scarce in South Africa, despite the country’s high HIV/AIDS burden. This study, therefore, aimed to determine the prevalence of XDR-AB colonization in neonates, the proportion who develop invasive infection, and the in-hospital outcomes and risk factors associated with colonization recurrent outbreaks in these neonatal units.

## Methods

### Study setting

The study was conducted at Rahima Moosa Mother and Child Hospital (RMMCH), a large academic regional hospital in Johannesburg, South Africa. The hospital has 2,300 neonatal admissions annually and capacity for 61 neonatal beds. There are 10 NICU beds, 35 intermediate care beds, and 16 kangaroo mother care beds. The hospital also functions as a training center for medical students, residents, and pediatricians.

A retrospective case–control study was conducted in the intermediate neonatal ward and the NICU on neonates admitted from 1 June 2020 to 31 December 2022. The study period was chosen because of the availability of medical records. This research was approved by the Human Research Ethics Committee (HREC), University of Witwatersrand, Johannesburg (Approval number M23092; see Additional file 2).

#### Definitions

XDR-AB is an *A. baumannii* isolate that is non-susceptible to at least one agent in all but two or fewer antimicrobial categories.^17^

XDR-AB colonization refers to having XDR-AB cultured from a rectal or skin screening swab taken after 72 hours of admission.^3^

The XDR-AB non-colonized neonate was defined as one who had a negative XDR-AB screening swabs during admission.

Antibiotic exposure indicates that a patient received at least one antibiotic before a positive screening swab or culture.

Invasive XDR-AB disease is defined as the first positive XDR-AB culture obtained from a sterile site such as blood or cerebrospinal fluid (CSF).

### Selection and description of participants

Screening for XDR-AB colonization was initiated in March 2019 during an outbreak in the unit, but routine surveillance continued even after the outbreak was declared over in July 2019. All neonates in the neonatal intensive care unit were screened. In the other neonatal wards, only XDR *A. baumannii*-exposed neonates were screened, *i.e*. if one neonate in the cubicle had XDR *A. baumannii* infection, then the rest of the neonates in the same cubicle would be screened and cohorted according to their screening swab results. Each neonate had one rectal swab and one skin screening swab (the skin swab was used to swab both the groin and the axillae).

The inclusion criteria were as follows: all admitted neonates (less than or equal to 28 d) admitted at RMMCH intermediate neonatal ward and NICU who had an XDR-AB screening swab (skin and/or rectal swab) between 1 June 2020 and 31 December 2022. Only the first positive screening swab was included, and subsequent XDR-AB screening swabs were not considered. The exclusion criterion was patients whose medical records or clinical data were missing.

### Processing of screening swabs

The identification of XDR-AB from a screening swab, blood culture, and cerebrospinal fluid culture in the laboratory was done by planting the sample directly onto MacConkey agar and incubating agar plates aerobically for 24 hours. Non-lactose fermenting colonies were isolated and identified using either VITEK® 2 system (bioMérieux, Marcy-l’Étoile, France) or API20E ID strips (bioMérieux, Marcy-l’Étoile, France). Antibiotic susceptibility testing was done either using VITEK® 2 system (bioMérieux, Marcy-l’Étoile, France) or manually using the Kirby-Bauer disk diffusion method.

### Data collection

The laboratory data of all XDR-AB screening swabs (rectal and/or skin) were retrospectively extracted from the NHLS Corporate Data Warehouse (CDW) database to determine prevalence of colonization. Invasive XDR-AB infections were identified from the first sterile-site specimen, such as blood and/or CSF that cultured XDR-AB. Clinical information and in-hospital outcomes were obtained from the RMMCH neonatal database. All different datasets were cleaned, merged using unique patient identifiers in STATA and anonymised before analysis. The independent variables were XDR-AB screening results, sex, birth weight, gestational age, age at testing, HIV exposure and HIV PCR results, surgical history, presence of invasive devices, ICU admission and duration of ICU stay, antibiotic exposure, diagnosis at admission, mode of delivery, and type of feeding. The dependent variables were in-hospital outcomes and the presence of XDR-AB invasive disease.

### Statistical analysis

Descriptive statistics were used to summarize the baseline demographics and clinical characteristics of the participants. Continuous variables were assessed for normality using the kernel density distribution and are presented as medians and interquartile ranges (IQRs). Age was analyzed with the Mann–Whitney *U* test after assessing the normality of distribution. Categorical variables are presented as frequencies (numbers, percentages) and were compared using the Pearson χ^2^ test. To determine the prevalence of XDR-AB colonization in neonates, frequencies and percentages were used. To assess the prevalence of invasive XDR-AB infections in colonized neonates, frequency and percentages were used and were compared using the Pearson χ^2^ test. Stepwise logistic regression was used to determine the factors significantly associated with XDR-AB colonization. A probability value of 0.15 was used for selection. All the models were assessed for goodness of fit. Statistical analyses were performed using STATA version 18.0 software (StataCorp. 2023. Stata Statistical Software: Release 14. College Station, TX: StataCorp LP). A *P* value of <.05 was considered to indicate statistical significance.

## Results

**Figure 1.**
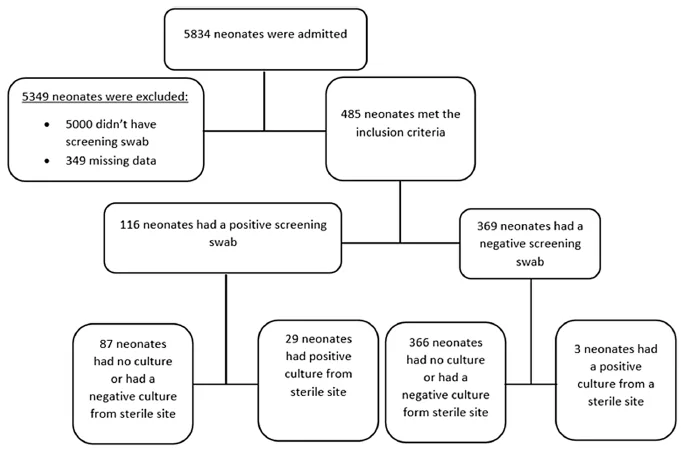
Flow diagram of participants study inclusion.

We included medical and laboratory results of 485 participants in this study. Figure 1 illustrates the flow of participants inclusion into the study.

### Demographics of the study population and prevalence of XDR-AB colonization

This study included 485 participants. There were 227 (46.8%) female participants, median age of the participants was 4 days (IQR: 2–7 d). The majority of participants, 151 (31.1%) were within the 28–32 weeks’ gestational age category, and most neonates (206; 42.5%) had birth weight of >2500 g. Most neonates 386 (79.6%) had no surgical history, and about 254 (52.4%) neonates were exclusively fed with breastmilk. Previous antibiotic exposure was reported in 291 (60.0%) neonates. The majority of neonates, 355 (73.2%) were admitted in intensive critical care. Among the participants, 200 (41.2%) participants were delivered by cesarean section, and most participants 292 (60.2%) were HIV-unexposed (Table 1). Among the 485 participants, 116 (23.9%) were colonized with XDR-AB, whereas 369 (76.1%) were not. The gender and age distribution was similar across both groups.

**Table 1.** Demographic description and prevalence of XDR-AB colonization in neonates at RMMCH Table 1 long description.

	XDR-AB colonization			
	Non-colonised	Colonised	Total	*P*-value
**Number of participants**	369 (76.1%)	116 (23.9%)	485 (100.0%)	
**Age in days**	4.0 (2.0–7.0)	4.0 (2.9–7.0)	4.0 (2.0–7.0)	.941
**Sex**				.682
Female	173 (46.9%)	54 (46.6%)	227 (46.8%)	
Male	134 (36.3%)	46 (39.7%)	180 (37.1%)	
Unknown	62 (16.8%)	16 (13.8%)	78 (16.1%)	
**Gestational age**				.009
<28 weeks	13 (3.5%)	11 (9.5%)	24 (4.9%)	
28–32 weeks	111 (30.1%)	40 (34.5%)	151 (31.1%)	
33–37 weeks	72 (19.5%)	27 (23.3%)	99 (20.4%)	
>37 weeks	97 (26.3%)	16 (13.8%)	113 (23.3%)	
Unknown	76 (20.6%)	22 (19.0%)	98 (20.2%)	
**Birth weight (grams)**				.003
<1,000 g	22 (6.0%)	19 (16.4%)	41 (8.5%)	
1,000 g–1,500 g	81 (22.0%)	28 (24.1%)	109 (22.5%)	
1,500 g–2,500 g	101 (27.4%)	28 (24.1%)	129 (26.6%)	
>2,500 g	165 (44.7%)	41 (35.3%)	206 (42.5%)	
**Ventilation**				<.001
No	188 (50.9%)	39 (33.6%)	227 (46.8%)	
Yes	105 (28.5%)	55 (47.4%)	160 (33.0%)	
Unknown	76 (20.6%)	22 (19.0%)	98 (20.2%)	
**Surgical history**				.192
No	293 (79.4%)	93 (80.2%)	386 (79.6%)	
Yes	0 (0.0%)	1 (0.9%)	1 (0.2%)	
Unknown	76 (20.6%)	22 (19.0%)	98 (20.2%)	
**Type of feeding**				.765
Breast milk	190 (51.5%)	64 (55.2%)	254 (52.4%)	
Formula	12 (3.3%)	3 (2.6%)	15 (3.1%)	
Unknown	167 (45.3%)	49 (42.2%)	216 (44.5%)	
**ICU admission**				.615
No	101 (27.4%)	29 (25.0%)	130 (26.8%)	
Yes	268 (72.6%)	87 (75.0%)	355 (73.2%)	
**Antibiotic exposure**				.016
No	83 (22.5%)	13 (11.2%)	96 (19.8%)	
Yes	210 (56.9%)	81 (69.8%)	291 (60.0%)	
Unknown	76 (20.6%)	22 (19.0%)	98 (20.2%)	
**Admission diagnosis**				.033
Sepsis	9 (2.4%)	4 (3.4%)	13 (2.7%)	
CNS pathology	33 (8.9%)	12 (10.3%)	45 (9.3%)	
GIT pathology	27 (7.3%)	15 (12.9%)	42 (8.7%)	
Lung pathology	71 (19.2%)	33 (28.4%)	104 (21.4%)	
Cardiovascular pathology	7 (1.9%)	4 (3.4%)	11 (2.3%)	
Genetic disorder	1 (0.3%)	1 (0.9%)	2 (0.4%)	
Liver pathology	2 (0.5%)	0 (0.0%)	2 (0.4%)	
Unknown	219 (59.3%)	47 (40.5%)	266 (54.8%)	
**Mode of delivery**				.301
Cesarean section	145 (39.3%)	55 (47.4%)	200 (41.2%)	
Normal vaginal delivery	135 (36.6%)	37 (31.9%)	172 (35.5%)	
Unknown	89 (24.1%)	24 (20.7%)	113 (23.3%)	
**HIV exposure**				.289
No	228 (61.8%)	64 (55.2%)	292 (60.2 %)	
Yes	55 (14.9%)	24 (20.7%)	79 (16.3%)	
Unknown	86 (23.3%)	28 (24.1%)	114 (23.5%)	

There were 32 neonates in total (both colonized and non-colonized) from the entire study who subsequently developed XDR-AB positive cultures from sterile sites. Invasive disease was significantly more common among neonates colonized with XDR-AB. Among the colonized neonates, 29 (25%) progressed to invasive XDR-AB disease, while 3 (0.81%) non-colonized neonates developed invasive XDR-AB disease. The odds of invasive disease in the colonized neonates were 41 times higher than the odds of invasive disease in non-colonized neonates (*P* value <.001, 95% CI: 12.07–211.26) (Table 2).

**Table 2.** Prevalence of invasive XDR-AB infections in colonised and non-colonised neonates Table 2 long description.

	Invasive disease	Non-invasive disease	Total (n)
**Colonised neonates (N, %)**	29 (25%)	87 (75%)	116
**Non-colonised neonates (N, %)**	3 (0.81%)	366 (99.19%)	369
Total	**32**	**453**	**485**

In-hospital mortality was significantly higher in XDR-AB colonized neonates compared to non-colonized neonates (10.34% vs 1.36%, respectively, *P* = .001), with strong evidence indicating a significant association between XDR-AB colonization and in-hospital mortality. Colonized neonates had 7.41 times higher odds of dying compared to the non-colonized group (*P* = .001, 95% CI: 2.25–24.38). Other in-hospital outcomes were not significantly associated with colonization status (Table 3).

**Table 3.** Relationships between in-hospital outcomes in colonised and non-colonised neonates Table 3 long description.

Outcome	Colonised neonates (n = 116)	Non-colonised neonates (n = 369)	Total	OR	*P*	95% Cl
**Died**	12 (10.34%)	5 (1.36%)	17 (3.51%)	7.41	.001	2.25–24.38
**Discharged home**	55 (47.41%)	177 (47.97%)	232 (47,84%)	0.69	.234	0.38–1.27
**Transfer to other wards**	17 (14.66%)	79 (21.41%)	96 (19.79%)	0.75	.39	0.39–1.44
**Transfer to another facility**	3 (2.59%)	8 (2.17%)	11 (2.27%)	1.17	.28	0.28–4.93
**Unknown**	29 (25%)	100 (27.10%)	129 (26.60%)	0.93	.792	0.23–0.42

Little MCAR test was significant (*P* < .001), indicating that the missing data were not completely at random. Despite considering multiple amputation for variables with approximately 20% missing data, complete-case analysis was used because 80% of observations were complete and missingness was limited to a few variables, making this a practical and transparent approach. In the final logistic regression model, only four variables remained independently associated with colonization: antibiotic exposure, gestational age, invasive ventilation and birth weight. In the univariate analysis, the variables had significant association with colonization but in the multivariate analysis they were influenced by confounding factors (Table 4).

**Table 4.** Logistic regression model accounting for confounding factors associated with XDR-AB colonization Table 4 long description.

		XDR-AB Colonization					
		Un-adjusted			Adjusted		
Factors	Label	OR	*P*	95% CI	OR	*P*	95% CI
**Antibiotic exposure**	No	baseline					
	Yes	2.46	.006	1.30–4.66	1.87	.117	0.85–4.08
**Birth weight**	<1,000 g	Ref					
	1,000 g–1,500 g	0.43	.017	0.19–0.85	0.43	.105	0.16–1.19
	1,500 g–2,500 g	0.32	.003	0.15–0.67	0.47	.169	0.16–1.38
	>2,500 g	0.29	.001	0.14–0.58	0.58	.428	0.15–2.21
**Mechanical ventilation**	No	baseline					
	Yes	2.53	.000	1.57–4.06	1.44	.213	0.81–2.60
**Gestational age**	<28 weeks	baseline					
	28–32 weeks	0.43	.057	0.18–1.03	0.65	.55	0.16–2.58
	33–37 weeks	0.44	.082	0.18–1.10	0.80	.766	0.18–3.54
	>37 weeks	0.20	.001	0.07–0.51	0.38	.245	0.72–1.96

## Discussion

This study assessed the prevalence of XDR-AB colonization and its progression to invasive disease among neonates at RMMCH from 1 June 2020 to 31 December 2022. The guts of neonates are colonized with XDR-AB from their mothers and the environment shortly after birth.^3^ The median age of the neonates in this study was 4 days, indicating colonization early in life. RMMCH neonatal wards showed a high burden of XDR-AB colonization at 23.9%, compared with 12%–21.4% reported in the literature, highlighting the significant problem of HAIs in neonatal wards.^3,13–15^ Moreover, this high prevalence reflects the high multidrug-resistant organism (MDRO) burden observed in LMICs and indicates ongoing infection prevention and control challenges within this unit. The high prevalence may also be explained by the fact that most neonates were admitted from the intensive care unit.

Following colonization, XDR-AB can translocate from the gut into the bloodstream, leading to invasive disease due to immature defence mechanisms in neonates.^3^ This study found a significant association between colonization and an increased risk of invasive disease (OR 41). Meschiari *et al*. demonstrated that the risk of subsequent XDR-AB invasive disease increases more than eightfold with colonization.^18^ Other studies reported that approximately 7.9%–25% of colonized neonates in the NICU develop invasive disease, including bloodstream infections.^3,13,16^

The mortality rate among colonized neonates at RMMCH was 10.34%, which was lower than the 26% reported in the previous literature.^3^ This rate aligns with the findings of Ferreira *et al.* who reported a mortality rate of 12.8% among neonates colonized with multidrug-resistant organisms.^19^ Although our study did not assess mortality among neonates with XDR-AB invasive disease, previous research has shown significantly higher mortality in such infections, reaching 75%; although, the study sample size was small.^3^ Importantly, most South African studies have focused on infected neonates, with limited data on outcomes in colonized infants.

Antibiotic exposure, mechanical ventilation, gestational age and birth weight initially appeared to be associated with colonization in the current study, although the association diminished after adjusting for confounding variables. Previous studies that identified a younger gestational age as a significant risk factor.^15^ Similarly, invasive ventilation for more than 5 days was found to increased susceptibility to colonization due to mucosal damage and alterations in air pressure and haemodynamics.^11,13,18^ Previous studies have shown that carbapenems exposure was linked to XDR-AB colonization (OR 5.39), whereas other studies found no significant associations with commonly used neonatal antibiotic regimens such as ampicillin-gentamicin or ceftriaxone-gentamicin.^15,18^

The mode of delivery showed marginal significance for intestinal colonization but lost significance in the final model (15). Moreover, delivery by cesarean section has previously been identified as a significant risk factor for early colonization (*P* = .04).^13^ Our study did not evaluate the association between HIV exposure or infection with *A. baumannii* colonization or infection due to missingness. Previous studies have found that HIV exposure or infection is not a significant risk factor for colonization in adults and infants.^20^

## Limitations of the study and strengths of the study

The limitations of this study include its single-center retrospective design within a population that has a high burden of XDR-AB. This may limit generalizability to other contexts. It was not possible to assess infection prevention control-related risk factor strategies. The wide confidence interval for XDR-AB colonization leading to XDR-AB invasive disease, suggests that a larger sample size would have provided more certainty. There were substantial amounts of missing data, for example gender, gestational age and a substantial number of participant outcomes were unknown. In the final model; mode of delivery, HIV PCR, HIV exposure, and admission diagnosis could not be evaluated further due to significant missingness. Only neonates who subsequently has positive sterile site cultures were evaluated it was difficult to interpret the value of negative sterile site cultures in this study cohort because of confounders for example inadequate amount of blood, timing of phlebotomy for blood cultures, concurrent antibiotic treatment that can lead to “false negative” and difficult to obtain the total number of sterile cultures collected. These gaps in data could impact the validity and accuracy of the study results. Additionally, the absence of clinical data on the use of invasive devices (other than mechanical ventilators), mixed feeds, and length of stay limited the assessment of their impact on XDR-AB colonization. Long-term mortality following colonization could not be assessed due to the lack of postdischarge follow-up data. Despite these limitations, the study’s strength lies in its focus on the risk factors for XDR-AB colonization, eliminating potential confounding from invasive disease. This approach allowed for a detailed investigation of factors associated with XDR-AB colonization and its associated risk factors in neonates within the hospital.

## Conclusion

The study demonstrated that most neonates in these units were colonized with XDR-AB at an early age, with a notably high prevalence of XDR-AB colonization. XDR-AB colonization significantly increased the risk of developing invasive disease among neonates and in-hospital mortality was notably higher in the colonized group as compared to non-colonized neonates. These findings highlight that early identification of colonized neonates could predict neonates at risk of developing invasive XDR-AB infection and subsequent mortality. Based on the findings, we recommend XDR-AB screening in this unit, for neonates who are mechanically ventilated, together with heightened vigilance for invasive disease when sepsis occurs in colonized neonates. Empiric antibiotic treatment should be tailored to include coverage for XDR-AB in colonized neonates, given the high mortality rate associated with invasive infection.

## Manuscript preparation

During the preparation of this manuscript, the authors used M365 Copilot, model GPT-5 chat to assist with language editing and grammar correction. All generated content was reviewed by the authors after using the tool. The authors take full responsibility for the final version of the manuscript.

## Research transparency and reproducibility

The de-identified datasets used and/or analyzed during the current study will made on reasonable request.

## Conference presentations

This study was presented as a poster at the ESCMID conference (April 2025, Austria) and subsequently at the ICAN conference in Cape Town (June 2025).

## Table 1. Long description

A table with 16 rows and 8 columns comparing demographic data and XDR-AB colonization in neonates. Columns include Non-colonised, Colonised, Total, and P-value. Row labels include Number of participants, Age in days, Sex, Gestational age, Birth weight, Ventilation, Surgical history, Type of feeding, ICU admission, Antibiotic exposure, Admission diagnosis, Mode of delivery, and HIV exposure. Row 1: Non-colonised, 369 (76.1%); Colonised, 116 (23.9%); Total, 485 (100.0%); P-value, N/A. Row 2: Non-colonised, 4.0 (2.0-7.0); Colonised, 4.0 (2.9-7.0); Total, 4.0 (2.0-7.0); P-value, 0.941. Row 3: Non-colonised, Female 173 (46.9%), Male 134 (36.3%), Unknown 62 (16.8%); Colonised, Female 54 (46.6%), Male 46 (39.7%), Unknown 16 (13.8%); Total, Female 227 (46.8%), Male 180 (37.1%), Unknown 78 (16.1%); P-value, 0.682. Row 4: Non-colonised, <28 weeks 13 (3.5%), 28-32 weeks 111 (30.1%), 33-37 weeks 72 (19.5%), >37 weeks 97 (26.3%), Unknown 76 (20.6%); Colonised, <28 weeks 11 (9.5%), 28-32 weeks 40 (34.5%), 33-37 weeks 27 (23.3%), >37 weeks 16 (13.8%), Unknown 22 (19.0%); Total, <28 weeks 24 (4.9%), 28-32 weeks 151 (31.1%), 33-37 weeks 99 (20.4%), >37 weeks 113 (23.3%), Unknown 98 (20.2%); P-value, 0.009. Row 5: Non-colonised, <1,000 g 22 (6.0%), 1,000-1,500 g 81 (22.0%), 1,500-2,500 g 101 (27.4%), >2,500 g 165 (44.7%); Colonised, <1,000 g 19 (16.4%), 1,000-1,500 g 28 (24.1%), 1,500-2,500 g 28 (24.1%), >2,500 g 41 (35.3%); Total, <1,000 g 41 (8.5%), 1,000-1,500 g 109 (22.5%), 1,500-2,500 g 129 (26.6%), >2,500 g 206 (42.5%); P-value, 0.003. Row 6: Non-colonised, No 188 (50.9%), Yes 105 (28.5%), Unknown 76 (20.6%); Colonised, No 39 (33.6%), Yes 55 (47.4%), Unknown 22 (19.0%); Total, No 227 (46.8%), Yes 160 (33.0%), Unknown 98 (20.2%); P-value, <0.001. Row 7: Non-colonised, No 293 (79.4%), Yes 0 (0.0%), Unknown 76 (20.6%); Colonised, No 93 (80.2%), Yes 1 (0.9%), Unknown 22 (19.0%); Total, No 386 (79.6%), Yes 1 (0.2%), Unknown 98 (20.2%); P-value, 0.192. Row 8: Non-colonised, Breast milk 190 (51.5%), Formula 12 (3.3%), Unknown 167 (45.3%); Colonised, Breast milk 64 (55.2%), Formula 3 (2.6%), Unknown 49 (42.2%); Total, Breast milk 254 (52.4%), Formula 15 (3.1%), Unknown 216 (44.5%); P-value, 0.765. Row 9: Non-colonised, No 101 (27.4%), Yes 268 (72.6%); Colonised, No 29 (25.0%), Yes 87 (75.0%); Total, No 130 (26.8%), Yes 355 (73.2%); P-value, 0.615. Row 10: Non-colonised, No 83 (22.5%), Yes 210 (56.9%), Unknown 76 (20.6%); Colonised, No 13 (11.2%), Yes 81 (69.8%), Unknown 22 (19.0%); Total, No 96 (19.8%), Yes 291 (60.0%), Unknown 98 (20.2%); P-value, 0.016. Row 11: Non-colonised, Sepsis 9 (2.4%), CNS pathology 33 (8.9%), GIT pathology 27 (7.3%), Lung pathology 71 (19.2%), Cardiovascular pathology 7 (1.9%), Genetic disorder 1 (0.3%), Liver pathology 2 (0.5%), Unknown 219 (59.3%); Colonised, Sepsis 4 (3.4%), CNS pathology 12 (10.3%), GIT pathology 15 (12.9%), Lung pathology 33 (28.4%), Cardiovascular pathology 4 (3.4%), Genetic disorder 1 (0.9%), Liver pathology 0 (0.0%), Unknown 47 (40.5%); Total, Sepsis 13 (2.7%), CNS pathology 45 (9.3%), GIT pathology 42 (8.7%), Lung pathology 104 (21.4%), Cardiovascular pathology 11 (2.3%), Genetic disorder 2 (0.4%), Liver pathology 2 (0.4%), Unknown 266 (54.8%); P-value, 0.033. Row 12: Non-colonised, Cesarean section 145 (39.3%), Normal vaginal delivery 135 (36.6%), Unknown 89 (24.1%); Colonised, Cesarean section 55 (47.4%), Normal vaginal delivery 37 (31.9%), Unknown 24 (20.7%); Total, Cesarean section 200 (41.2%), Normal vaginal delivery 172 (35.5%), Unknown 113 (23.3%); P-value, 0.301. Row 13: Non-colonised, No 228 (61.8%), Yes 55 (14.9%), Unknown 86 (23.3%); Colonised, No 64 (55.2%), Yes 24 (20.7%), Unknown 28 (24.1%); Total, No 292 (60.2%), Yes 79 (16.3%), Unknown 114 (23.5%); P-value, 0.289.

Navigate back to Table 1..

## Table 2. Long description

A table comparing the prevalence of invasive XDR-AB infections in colonized and non-colonized neonates. The table has four rows and three columns. The columns are labeled ʼInvasive diseaseʼ, ʼNon-invasive diseaseʼ, and ʼTotal (n)ʼ. The rows are labeled ʼColonised neonates (N, %)ʼ and ʼNon-colonised neonates (N, %)ʼ. Row 1: Invasive disease, 29 (25%); Non-invasive disease, 87 (75%); Total (n), 116. Row 2: Invasive disease, 3 (0.81%); Non-invasive disease, 366 (99.19%); Total (n), 369. Row 3: Invasive disease, 32; Non-invasive disease, 453; Total, 485.

Navigate back to Table 2..

## Table 3. Long description

The table presents a comparison of in-hospital outcomes between colonized and non-colonized neonates. It has six rows and seven columns. The columns are labeled Outcome, Colonised neonates (n = 116), Non-colonised neonates (n = 369), Total, OR, P, and 95% CI. The rows are labeled Did, Discharged home, Transfer to other wards, Transfer to another facility, and Unknown. Row 1: Did, 12(10.34 percent), 5(1.36 percent), 17(3.51 percent), 7.41, .001, 2.25-24.38. Row 2: Discharged home, 55(47.41 percent), 177(47.97 percent), 232(47.84 percent), 0.69, .234, 0.38-1.27. Row 3: Transfer to other wards, 17(14.66 percent), 79(21.41 percent), 96(19.79 percent), 0.75, .39, 0.39-1.44. Row 4: Transfer to another facility, 3(2.59 percent), 8(2.17 percent), 11(2.27 percent), 1.17, .28, 0.28-4.93. Row 5: Unknown, 29(25 percent), 100(27.10 percent), 129(26.60 percent), 0.93, .792, 0.23-0.42.

Navigate back to Table 3..

## Table 4. Long description

A table with four rows and five columns comparing factors associated with XDR-AB colonization. The table includes un-adjusted and adjusted odds ratios (OR), P values, and 95% confidence intervals (CI) for each factor. The factors listed are antibiotic exposure, birth weight, mechanical ventilation, and gestational age. For antibiotic exposure, the un-adjusted OR is 2.46 with a P value of 0.006 and a 95% CI of 1.30-4.66, while the adjusted OR is 1.87 with a P value of 0.117 and a 95% CI of 0.85-4.08. For birth weight, the categories are <1,000 g, 1,000-1,500 g, 1,500-2,500 g, and >2,500 g, with varying un-adjusted and adjusted ORs, P values, and 95% CIs. For mechanical ventilation, the un-adjusted OR is 2.53 with a P value of 0.000 and a 95% CI of 1.57-4.06, while the adjusted OR is 1.44 with a P value of 0.213 and a 95% CI of 0.81-2.60. For gestational age, the categories are <28 weeks, 28-32 weeks, 33-37 weeks, and >37 weeks, with varying un-adjusted and adjusted ORs, P values, and 95% CIs.

Navigate back to Table 4..
